# A prospective observational study evaluating the short-term effectiveness of residential care for adolescents as “service as usual”: A study protocol

**DOI:** 10.1371/journal.pone.0347409

**Published:** 2026-04-24

**Authors:** Pia K. Eriksson, Elina Aaltio, Mikko Aaltonen, Taina Laajasalo

**Affiliations:** 1 Faculty of Social Sciences, University of Helsinki, Helsinki, Finland; 2 Safety and Protection, Child Welfare Services, Finnish Institute for Health and Welfare, Helsinki, Finland; 3 Faculty of Social Sciences, Tampere University, Tampere, Finland; 4 Law School, Faculty of Social Sciences and Business Studies, University of Eastern Finland, Joensuu, Finland; IRCCS Medea: Istituto di Ricovero e Cura a Carattere Scientifico Eugenio Medea, ITALY

## Abstract

Previous research indicates worse outcomes for children and young people in out-of-home care compared to their peers. To improve the quality of current residential care, research is needed to deepen our understanding of the key factors and mechanisms that explain the effectiveness of residential care on child level. The aim of this study is to evaluate the short-term (i.e., during care) effectiveness of residential care as a child welfare intervention by different service providers. The study includes two pre-specified primary child-level outcomes 1) psychosocial functioning, assessed through the Strengths and Difficulties Questionnaires (SDQ) including both total difficulties scores and subscales and 2) attainment of individual goals of the child. Secondary outcome measures include improved experiences of emotional warmth and safety (CEWSS-A). Further associations between key characteristics of the residential care unit, the child and outcomes are assessed. The data consist of longitudinal survey data collected from 12–17-year-old children and staff in a total sample of public and private residential care units offering specialized care in three wellbeing services countries in Finland. The baseline (T0) data is collected between 1.4.2025–31.8.2026 with a follow up of 6 and 12 months for each child (T1 and T2). The data is primarily analysed with linear mixed models. Findings explore the short-term effectiveness and change mechanisms of residential care as “service as usual” to understand how it should be organised and produced to improve its ability to meet aims during care. The study design embraces the complexity and changeability on different levels of the residential care setting.

## Introduction

This study will assess the short-term effectiveness of residential care (RC) for children and youth as an out-of-home care (OHC) service. The prospective observational study evaluates child-level outcomes of RC as a child welfare “service as usual” for 12–17-year-old adolescents over twelve months.

Previous research indicates worse outcomes for children having lived in OHC compared to their peers. Studies comparing outcomes in Nordic and European countries [1–3] have found that, in light of register-based outcome data, children in care are worse off compared to the general population in terms of many indicators such as educational attainment, health, mortality and delinquency. Further comparisons of outcomes of RC and foster family care [4–7] and different kinds of family-based care [8,9] show that RC has least favourable outcomes. As comparisons between children in different types of OHC have been made, less attention has been paid to the RC setting specifically.

In Finland, 1.6 percent of all children under 18 were placed in OHC during the year 2024. Of these 17,100 children 49% (8405) had been living in RC for a shorter or longer period during the year. Children placed in long-term RC, are primarily teenagers and the target group of this study. As of December 31, 2024, the total number of 12–17-year-olds in RC in Finland was 4,209. The number increases progressively with age, starting from 290 individuals aged 12 and reaching a peak of 1,000 individuals aged 17. In Finland OHC covers over 74% of child welfare costs (approx. 1 billion euros), RC being the costliest type of OHC accounting for 672 million euro yearly. [10,11] Still, very little is known about the effectiveness of the RC offered.

A scoping review [12] on the short-term (during care) outcomes of RC found only 17 original studies focusing on outcomes during the RC placement. In these studies, the changes in the wellbeing or functioning of children and youth during care were evaluated from three perspectives: by assessing the outcomes of new innovations developed for RC, implemented interventions, or RC as service as usual. Eight studies on RC as service as usual suggested that most children benefit from the care, and some sub-groups more than others. However, many of these studies lacked a specific theory of change to inform the study and the interpretation of the findings. Studies on quality in RC suggest that main indicators of quality are related to the process of service delivery (e.g., relationship between staff and children, the involvement of family, child´s motivation) and the RC unit as a context (e.g., safety, home-likeness, social climate).

In Finland RC is provided by public and private non-profit and for-profit service providers. According to the legislation, the number of children in one unit must not exceed seven, and the maximum number of children in one building is 24. In the privately run care facilities, the number of children is approximately 18 [13] and in the public units of wellbeing services counties 11. Further units differ in the provision of in-house services, methods used, staff ratio and staff’s training [14]. All these characteristics may affect the effectiveness of the care. In previous research, conducted in Great Britain and the United States, some differences in quality deficits were found between public and private RC facilities [15,16]. In Finland, over 80% of RC units for children and youth are owned by private, mostly for-profit providers, a share which is higher than in other Nordic countries [13,17]. However, no comparison of the effectiveness of Finnish RC between different types of providers has been conducted. In Finland the Parliamentary Ombudsman has identified omissions both in public and private RC units during on-site investigations [see, e.g., 18]. To better understand what constitutes the mechanisms leading to effective RC on child-level, a more detailed analysis of its key features and prerequisites is needed.

### Residential care

In general, RC is considered the last option in the selection of child welfare services. Most of the child welfare services in Finland are voluntary in-home services aiming to support children and their families in their living environment as according to the Child Welfare Act (417/2007) these always are primary. A child can be removed from their home only if in-home services are not sufficient. There are three types of placement grounds 1) a temporary placement as part of in-home services based on the consent of the custodians and the child, 2) an emergency placement for up to sixty days and 3) a care order. A care order can be made if three conditions are met: the in-home services are not relevant or appropriate, the child’s health or development is at risk of being seriously endangered, and care order serves the child’s best interest. Care orders may be either voluntary or involuntary [for more details, see 19]. Voluntary removals and placements are made by social workers, and involuntary placements are treated as administrative judicial matters in administrative courts but the legal consequences upon decision are similar.

According to the Finnish legislation all placements into OHC are meant to be temporary, but in practice reunifications are rare as only 4% of care orders are discharged. Many children stay in care for several years: of all children removed from their homes during 2023, one fourth had been living in OHC for six years or more and one third at least half of their lives. OHC is mainly arranged as foster family care, in professional foster homes or as RC. Of for example 15-year-old teens in care, 39% are living in foster families or professional foster homes, 59% in RC and 2% in other way (e.g., independent supported housing). [10,11] Foster family care should always be considered before RC, but most teenagers are placed in RC due to their complex needs. The type and place for alternative care should be chosen based on the child’s individual needs, ensuring the child’s close social relations, continuation of the care, and if possible, considering child’s linguistic, cultural and religious background. However, in practice, social workers make many compromises due to the availability or costs of alternative care places or preferences of children and parents [20,21].

## Objectives

The objective of this study is to evaluate the short-term effectiveness of RC as a child welfare intervention for adolescents and to enhance understanding of the factors that affect child-level outcomes. Firstly, the associations between the type of provider (private, public) and child-level outcomes are analysed using SDQ (Strengths and Difficulties Questionnaire) and individual goal attainment of children as primary outcomes. As secondary outcomes experiences of emotional warmth and safety are assessed. Secondly, the mediating effect between child and unit level variables with child-level outcomes is investigated. To inform the study, a programme theory was formulated, in a collaborative process with researchers, experts, and adolescents and staff in RC units, to identify the context, key mechanisms, and desired outcomes of RC as child welfare intervention, and to formulate hypotheses explaining how the context (i.e., key characteristics of RC units and staff) may affect the working of these mechanisms.

The research questions are:

RQ1:

a) Are there changes in child-level outcomes at six and twelve-month follow-up?
b) Is the service provider type (public vs. private) associated with variation in child-level outcomes?

RQ2:

a) What is the mediating effect of social climate, treatment motivation and co-operation with parents on the child-level outcomes?
b) Is the service provider type associated with the mediating effect of social climate, treatment motivation and co-operation with parents on child-level outcomes?

**Fig 1:**
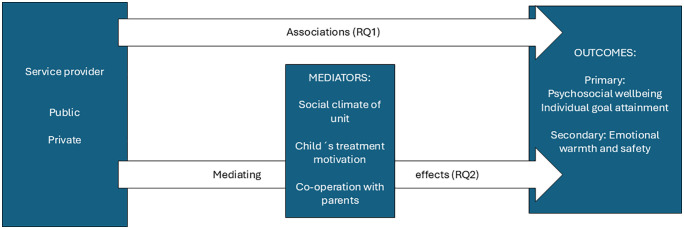
Overview of variable roles in research questions 1 and 2.

The roles of the variables in addressing the first two research questions are illustrated in Fig 1. The third research question is explorative and aims to enhance understanding of the mechanisms operating in residential care, drawing on a pre-defined program theory formulated within the project and addressed through the testing of the outlined hypothesis H1-H3.

RQ3: What additional relationships exist between unit and child-level variables and the outcomes observed?

H1 The level of SDQ-scores at baseline is the same across different units but the baseline and follow-up scores are associated with reason for placement and placement history of the child.

H2 Characteristics of the unit (size, staff and child turnover, unit cost, staff composition, and in-house services and practice framework used) is associated with the improvement in child-level outcomes.

H3 The effect of characteristics of the unit on the child level outcomes is mediated by level of collaboration with social worker, substance abuse and the child´s experience of trustful relationships, emotional warmth, and sense of safety.

## Materials and methods

### Study design

This is a prospective observational study on the outcomes of RC as “service as usual” across different service providers in three services wellbeing counties in Finland. The study is conducted as a follow-up survey study spanning 12 months, with both adolescents living and staff working in the RC units, as informants. Ethical approval was granted by the Institutional review board (IRB) of The Finnish Institute for Health and Welfare (THL/92/6.02.01/2025).

As the researchers acknowledge that RC is a complex intervention providing support for a heterogenous group of adolescents with various and often complex problems and needs, to inform the study design, a programme theory was formulated based on previous research findings and stakeholder perspectives in accordance with the UK Medical Research Council’s guidance for evaluating complex interventions [22]. The pre-defined programme theory identifies the targeted outcomes as well as the context and change mechanisms needed to provide effective RC. The programme theory has guided the selection of measures used in the data collection and is to be published separately.

### Study setting

In Finland, private RC units are classified into three levels (basic, specialised and demanding) during the procurement process. There are no national guidelines or legislation on these levels, but the staff ratio in specialised units is usually set to 1.3 per child. The features separating the different levels are connected to the capacity to answer to specific needs (substance abuse, mental health, antisocial behaviour, schooling etc.) and the staff ratio. This study includes units stating to provide long-term care on specialised level for 12–17-year-old adolescents. This level of care has been chosen to enhance understanding on the outcomes for adolescents with severe challenges in need of prolonged care that the systems struggle to meet. The specialised care also represents a markable larger proportion (52.7%) of the residential care units than the most demanding units (15.9%) [23]. Long-term has here been defined as the usual duration of care in the units lasting at least one year.

According to a list compiled for this research project based on the national register of private service providers by the National Supervisory Agency (Valvira) and information gathered from all 21 counties on public units in Finland, the number of units nationally was 426 in the end of 2024. According to a survey approximately 60% of these units offer services on specialized level [23]. This study takes place in approximately 40 of these RC units in three counties in Finland.

The study will not implement any additional intervention within these units but explores “service as usual”. The research participants are typical adolescents living in these units, and practitioners working there.

### Eligibility criteria

All RC units providing RC for the target population in three selected counties are included in the study. To be eligible to participate in the study, RC unit must provide specialised long-term care, usually with a staff ratio of 1.3/ child. Otherwise, no specific inclusion or exclusion criteria will be used, since the study aims to produce research information on typical RC facilities not especially well resourced, experienced or innovative.

Individual participants are recruited from the participating RC units. All practitioners working in the participating units and all children that meet the inclusion criteria, are asked to participate. Participation is voluntary and the children and staff members take part based on written informed consent. The size of units is small and the number of new residents in a given RC unit is difficult to predict and can be as low as one or two per year. Hence, all eligible children living in participating units during 1.4.2025–31.8.2026 are asked to participate until a sufficient sample (N = 300) is achieved. To be eligible for the study, the child needs to be between 12 and 17 years old at the baseline.

### Intervention

The intervention under study is RC as “service as usual”. While many documents such as legislation and national guidelines offer descriptions of the content and minimum standards for RC, no national consensus has been formulated to describe the core components, targeted outcomes or mechanisms that are expected to produce these outcomes. As for example Kazdin [24] and the Swedish Association of Local Authorities & Regions [25] have noted “service-as-usual” (SAU) interventions often lacks important details and have poorly described content and information on expected outcomes. The content in RC in terms of theoretical framework, practice models and methods is diverse [14,23].

### Outcomes

All outcomes are on child-level. As the aim of RC is multi-fold with both the objective to enhance the psychosocial wellbeing of the child but also offer a safe and nurturing living environment, both these outcomes are included in this study. The primary outcome measures are related to the psychosocial wellbeing and individual goal attainment of the child. The secondary outcome measure targets the experiences of emotional warmth and safety.

### Primary outcome measures

Changes in psychosocial wellbeing will be measured through **psychosocial problems and prosocial behaviour** with the Strengths and Difficulties Questionnaire (SDQ). SDQ is a widely used questionnaire assessing distress and prosocial skills [26] and consists of 25 items on 5 scales: emotional symptoms, conduct problems, hyperactivity/inattention, peer relationship problems and prosocial behaviour. The forms for 11–17-year-old children and practitioner are used, as in addition to the child completing the form also his/her key worker evaluates the child. In addition, in the follow-up form include one questions for use after an intervention was included “After moving into the RC unit, has your problems reduced or increased?” SDQ has widely been used in the context of OHC and showed good to moderate psychometric properties in various studies [27]. The measure is available in Finnish.

**Attainment of the child´s individual goals** for the placement is measured with a questionnaire tailored by the researchers. The key workers of the children state the main individual goals that are set in the official documentation of the child´s placement and evaluate their attainment at six- and twelve-month follow-up applying the same scale as recommended in the national documentation guidelines. In the guideline a three-point scale is used: the goal has not been achieved, the goal has partially been achieved, and the goal is completely achieved. Further the key worker of the child fills in a questionnaire tailored for this study with basic information on, e.g., the reason for and duration of the placement and needs of the child.

### Secondary outcome measure

The adolescent´s **experiences of safety and emotional warmth** will be measured by the Current Experiences of Warmth and Safeness Scale for Adolescents (CEWSS-A) [28]. CEWSS-A is an adaptation of a measure that was originally created to assess early memories of caring experiences [29] and adapted to measure current experiences of adolescents. The measure has shown adequate internal consistency and construct validity in relation to external variables [28]. As part of the study, the scale was translated to Finnish and adapted in the Finnish RC context [30] through the FACIT-method [e.g., 31].

### Mediating variables

**The social climate** in RC units is measured with Essen Climate Evaluation Schema (EssenCES) that has been used for example in forensic psychiatry and correctional institutions [e.g., 32, 33]. The EssenCES, developed at the University of Duisburg was chosen as it is one of the few designed to be answered by both residents and staff [34] and a Finnish translation was available. Minor adaptation of wording suiting the RC context (e.g., patient and ward) was made by the research team and piloted with eight children (4 girls and 4 boys) aged 11–17-years living in RC. Minor adjustments to wording were made based on the feedback of the children and two questions on possible perceived threats from staff in the unit were added. The same measure is filled out both by the children and the staff in participating units.

**The child´s own motivation** for change and working on problems is measured with the Adolescent Treatment Motivation Questionnaire (ATMQ) [35]. The measure has previously been used in Dutch RC settings for children and youth [e.g., 36] and include questions associated with both motivation and **trustful relationships** with staff. The 11-item measure has been translated and culturally adapted by the members of the research team, one external expert and one professional translator following the FACIT-method [e.g., 31]. This measure was also piloted with eight children aged 11–17-years living in RC. Small adjustments to wording were made based on the feedback of the children. Two general questions on the level of self-efficacy have been added to the questionnaire.

**Collaboration with the family** is measured with four tailored questions regarding the family. The attitudes of RC staff towards the families of origin in general is measured with a question added to the EssenCES for the staff. Further the child´s perception of the parent´s acceptance of their placement is measured with one added question on the ATMQ for the child. Finally, two questions on acceptance of and co-operation with the individual child´s family is completed by the key worker in the questionnaire on basic information on the child. All four questions are answered on a Likert-scale, and the mean score based on a sum-variable is used in the analysis.

To assess the validity and reliability of the measures translated and adapted to the Finnish RC context within this study (CEWSS-A, ATMQ, EssenCES) their basic psychometric properties are to be studied based on the data gathered (e.g., internal consistency, item performance and construct validity).

### Other variables

Other variables consist of data on the residential care units and child characteristics. The unit level data captures the situation in the unit during the time of data collection. It includes the amount of children and child turnover in unit, staff composition (level of education and occupational diversity), staff-to-child ratio and staff turnover, cost per child per day, services offered in-house (education, therapy, health care) and practice model or framework used in unit. The child characteristics include basic information (age, gender, mother tongue and birth country of child and parents) and child welfare related variables (reason for placement, placement history, substance abuse).

### Data collection

The data collection from the adolescents is made in-person in the selected units at T0 (baseline), T1 (6 months) and T2 (12 months). Two follow-up points have been designed to ensure the commitment and likelihood of adolescents participating in at least one of the follow-up surveys. A member of the research team will visit each unit personally and assist the children with the electronic surveys filled out on electronic tablets provided. The data collected from staff is conducted through electronic surveys distributed by e-mail. The measures and respondents at each point is presented in Table 1.

**Table 1:** Schedule of enrolment and assessment.

	T0	T1	T2
**Respondent and measure**	Baseline	6 months follow-up	12 months follow-up
**Child**			
SDQ	x	x	x
CEWSS-A	x	x	x
ATMQ	x	x	x
Tailored questionnaire	x	x	x
EssenCES	x		
**Key worker of child**			
Child´s basic information	x		
SDQ	x	x	x
Goal attainment	x	x	x
**All staff**			
EssenCES	x		
**Manager of the unit**			
Unit level data	x		x

#### Characteristics of care units.

The managers of the units selected for the study fill out an electronic survey on characteristics of the unit and the staff in the beginning and end of data collection.

#### Survey for practitioners and all children at baseline.

The whole staff and all children will fill out the EssenCES measure for social climate in the unit.

#### Child-level data.

Child-level data will be collected from children and their key workers in the unit over the course of two years, with a follow-up of one year for each child. All children and young people living or moving into the unit between 1.4.2025–31.8.2026 are asked to participate in the study. If the child or young person is willing to participate, both the practitioner and the adolescent will complete questionnaires. The practitioner’s questionnaire includes questions on child characteristics, the attainment of goals of the placement, level of collaboration with parents and social worker, and SDQ. The questionnaire for child or young person includes the following measures 1) SDQ, 2) CESSW-A, 3) ATMQ 4) tailored questions. If an adolescent moves from the RC unit during the study, follow‑up data from measurement points after the move are not collected due to restrictions associated with the research permits, which results in missing data. The data collection will be finished by 31.8.2027 and the results reported in 2027–2029.

All data is collected using electronic questionnaires with secure software (REDCap; Research Electronic Data Capture). Each participating unit is visited by one member of the research group at each measurement point. The researcher assists the children in completing the questionnaires on electronic tablets provided by the project and ensures anonymity, informed consent and voluntary participation.

All staff and manager questionnaires and reminders are distributed electronically.

### Sample size

As there are no estimates of effect size on which to base our sample size calculations, we have made some assumptions to determine the sample size necessary to detect change in the primary outcome measure SDQ.

The estimations are based on a meaningful change of five points in the mean of SDQ scores between T0 and T2. The sample size was estimated based on a power analysis for repeated-measures ANOVA comparing two equally sized groups at two different time points, essentially examining differences in changes in SDQ over time in different subgroups. The required sample sizes with 0.05 significance level, 0.8 power and three different effect sizes (eta squared, small effect 0.01, medium effect 0.06 and large effect 0.14) was calculated. The calculation also assumed a 0.5 correlation between the two time points. With these assumptions detecting a small effect would require a total sample of 197, whereas for medium-sized effect a sample of 33 individuals would suffice. Thus, the total sample size of 300 children is expected to include enough children that have moved into the unit less than three months before T0 (group A) that should allow us to at least detect larger than medium-sized effects, depending on the final size of the groups. The rest of the children belong to group B) of children having lived in the unit for more than three months at T0. The estimated size of group A) is 120 children and of group B) 180 children. Robustness of the detected associations will be tested using linear mixed models.

### Recruitment

Recruiting practitioners and young people to studies in children’s social care may face multiple challenges, which may be rooted in unfamiliarity with research methods [37]. While senior managers may value participating in research in general, it does not guarantee the cooperation or commitment of the practitioners in the care units. To achieve adequate participant enrolment, we seek to create good collaboration with managers and practitioners in all participating care units by visiting each unit personally to give information about the study and to motivate practitioners to participate themselves and recruit adolescents to the study. Following suggestions by Oakley et al [38], we focus on priority issues for participants, seek to have a clear scientific and policy rationale for selected study design, allow sufficient time for detailed discussions with research participants and be sensitive to their perspectives, and carefully pilot recruitment and informed consent procedures that explain the design in accessible ways. The children will be rewarded with a gift card of 10 euros to a grocery store at T0, T1 and T2.

Personal contact by the principal investigator and each unit has been made and the co-operation agreed upon. The unit is provided with information for the children, staff, guardians and the social worker in the county responsible for the child´s case. Children are informed about the study by the staff through leaflets distributed to all children and verbal information by the staff. Voluntary participation for all parties is stressed.

### Allocation

All the eligible units within the selected counties were recruited to the study forming a total sample from the geographical areas. The counties were chosen to represent a possibly equal number of units of both provider type categories. The adolescents living in the public units are residents in the three counties where the units are located, whereas the adolescents in the private units are residents from fourteen different counties, as children are placed in care across county borders.

No additional innovations or interventions will be implemented in the participating units; hence the study will not allocate units to treatment and control groups, nor are allocation concealment mechanisms needed. The main comparison is made between service provider type.

Individual participant within participating units will not be randomly selected. Instead, a total sample is taken in each participating county and unit based on pre-defined criteria.

### Data management

The data will be stored and handled in the Finnish Institute for Health and Welfare (THL) and University of Helsinki. The General Data Protection Regulation is upheld, and data are stored and handled accordingly. As the data includes special categories of personal data of a vulnerable population extra care will be used when handling the data. The data will be collected with secure software (REDCap) and stored in secure databases. The data will be pseudonymized before analysis, and the key will be stored separately from the data.

### Statistical analysis

Associations between outcomes and care in different types of units will be analysed using linear mixed models that are well-suited to analyzing within-individual change in outcomes over time. In its most basic form, the analysis compares within-individual change by type of unit. Importantly, these models can account for the multilevel structure of the data, where repeated measurements will be nested within children, who are in turn nested within care units. Random intercepts for both care unit and child will be included to account for clustering at these levels, while random slopes can also be used to assess the variability of individual-level change over time. The mediating effect of child- and unit-level variables on the outcomes is conducted with multiple regression including mediating step-by-step analysis. The moderating influence of unit-level variables on change can be further analyzed with intercepts-and-slopes-as-outcomes linear mixed models.

For the analysis the children will be divided into two groups (data sets) based on their situation at baseline: A) new residents having lived less than 3 months in the unit and B) residents having lived in the unit for a longer time. RQ1 (assessment of effectiveness) is evaluated based on data set A). The research questions RQ2 and RQ3 (association between different variables and the outcomes) are analysed on the total set of data (group A and B). Missing outcome data will be handled within the mixed-model framework under a missing at random assumption, whereas missing mediator or covariate data will be addressed using multiple imputation.

## Ethical considerations

### Research ethics approval and research permits

The study has been approved by the Institutional review board (IRB) of The Finnish Institute for Health and Welfare in March 2025.

Research permits have been applied for from all twelve public and private service providers of the RC units participating. Additionally permits have been obtained from all fourteen counties that are responsible for the cases of the children placed in the private RC units

### Protocol amendments

The study will be conducted in accordance with the protocol. If modifications are required, the research ethics committee will be notified, followed by granters of research permits, i.e., the service providers and the counties. Minor changes will be agreed upon by the research group.

### Consent

Informed consent is obtained from all research participants, i.e., practitioners and children in participating care units. Written consent is collected from the children at baseline and oral consent at six- and twelve-month follow-up. The staff in the units give their written consent in the electronic questionnaire they are answering.

According to Finnish guidelines for ethical review in human sciences, if the minor is 15 or older, their own consent is sufficient for participation in the research. According to the Finnish constitution (6 §, 3 mom.) and the UN convention on children’s rights (article 12) children have the right to express their views freely in all matters affecting themselves, in accordance with their age and maturity. Children in OHC are a vulnerable group entitled to special protection and are therefore often precluded from taking part in studies on their situation by different gatekeepers. After attaining ethical approval children aged 12–14-years are given the same opportunity to take part in the study with their own written informed consent, as their older peers in the same RC units. Legal guardians (parents) and the social worker responsible for the child´s case, are informed about the study. Further special attention is paid to informing children individually by the research team both in writing and verbally to ensure informed consent.

### Confidentiality

All personal information will be collected with secure software (REDCap) and stored in secure databases. Data will be shared only with the research team, and with researchers involved in possible follow-up studies. All researchers are asked to sign a confidentiality agreement.

### Access to data

Only members of the research team have access to the dataset.

### Dissemination policy

The main findings will be reported and offered to be published in peer-reviewed journals regardless of the significance of the findings. The findings will be communicated to research participants, decision-makers, and the public via a seminar and a low threshold publication, e.g., policy brief. Along with the main findings, secondary analysis will be prepared. Since the data involves sensitive personal data, the data will not be shared.

Authorship eligibility guidelines will be applied.

## Discussion

This is to our knowledge the first attempt to do a large-scale study on the short-term outcomes of RC for adolescents in Finland. The intervention studied (service as usual) is much debated since both research on long-term outcomes (e.g., 2) and practice messages indicate that the aim of RC as a provider of a safe environment and enhancer of wellbeing is not always reached. Therefore, the findings have important practice implications not only in the delivery of RC services but also the organization of OHC services. As there is a lack of knowledge on how RC services answers to the needs of the children and how service as usual is contributing to positive outcomes for children and youth this study is not an intervention study adding elements to the setting, but the evaluation of effectiveness is based on comparison between different service providers.

The design of the study has posed many challenges as the RC field is scattered and diverse. The RC units are small, and the majority is run by private producers making access challenging. The level of care is not defined nationally and here the staff ratio and the units own definition has been the determinant of “specialised” units. RC duration varies greatly, and children are moved between units and their homes. The turn-over in children in the units is difficult to predict. The observational study aims to embrace the complexity of the service and the constant discontinuity in the lives of both staff and children in care since these cannot be controlled.

As research lacks and national statistics are insufficient, we do not have basic information on for example the different needs and challenges of the children at intake into different units. An ideal follow-up of the adolescents would have considered their individual placement duration and followed them from the intake to the exit from the unit, which is not possible within this study. Therefore, the follow-up time has been determined to twelve months. As a comparison is based on the precondition that the intervention (RC as service as usual) is similar in units run by different providers, based on the lack of evidence of the main content of the services offered being considerably different, we assume that the content is more similar than different in the two types of units. By allocating children into two groups for analysis, those who have recently moved into the unit (group A) and those who have lived there for a longer period (group B), we aim to obtain a large enough sample for the analysis, considering the hardship to estimate the participation rate among children and the dropout rate during the follow-up. The aim is to obtain a sufficient number of children having completed all three questionnaires (T0, T1 and T2) but acknowledging the rapid changes in the lives of children in OHC strategies for analysis can be modified if necessary.

The study will contribute to our understanding on how RC answers to the child level outcomes for RC placements and how different mechanisms contribute to positive or negative developments. Findings will also be the first in Finland to show whether there is a difference in the outcomes and explaining variables between different service providers. Also, the study is to our knowledge rare in an international perspective. Though in-home and family-based services are primary responses in child welfare, there is always going to be a proportion of adolescents in need of RC. As RC is the most expensive type of OHC and a placement of the child into RC an intrusive intervention into basic human rights and the personal sphere of families, the study of the effectiveness of the care offered is justified and important.
